# Characteristics of adults with type 1 diabetes and treatment-resistant problematic hypoglycaemia: a baseline analysis from the HARPdoc RCT

**DOI:** 10.1007/s00125-022-05679-5

**Published:** 2022-03-24

**Authors:** Peter Jacob, Laura Potts, Rory H. Maclean, Nicole de Zoysa, Helen Rogers, Linda Gonder-Frederick, Emma L. Smith, Dulmini Kariyawasam, Augustin Brooks, Simon Heller, Elena Toschi, Mike Kendall, Ioannis Bakolis, Pratik Choudhary, Kimberley Goldsmith, Stephanie A. Amiel, for the HARPdoc study group

**Affiliations:** 1 Department of Diabetes, Faculty of Life Sciences, King’s College London, London, UK; 2 Department of Biostatistics & Health Informatics, King’s College London, Institute of Psychiatry, Psychology and Neuroscience, London, UK; 3 Centre for Implementation Science, Health Services and Population Research Department, King’s College London, Institute of Psychiatry, Psychology and Neuroscience, London, UK; 4 King’s College Hospital NHS Foundation Trust, London, UK; 5 Centre for Diabetes Technology, Department of Psychiatry and Neurobehavioral Sciences, University of Virginia, Charlottesville, VA USA; 6 Guy’s and St Thomas’ Hospital, London, UK; 7 University Hospitals Dorset NHS Foundation Trust, Bournemouth, UK; 8 University of Sheffield, Sheffield, UK; 9 Joslin Diabetes Centre, Harvard Medical School, Boston, MA USA; 10 HARPdoc Patient Group, Department of Diabetes, King’s College London, London, UK; 11 University of Leicester, Leicester, UK

**Keywords:** Cognitive barriers, Hypoglycaemia, Hypoglycaemia fear, Impaired awareness of hypoglycaemia

## Abstract

**Aims/hypothesis:**

Problematic hypoglycaemia still complicates insulin therapy for some with type 1 diabetes. This study describes baseline emotional, cognitive and behavioural characteristics in participants in the HARPdoc trial, which evaluates a novel intervention for treatment-resistant problematic hypoglycaemia.

**Methods:**

We documented a cross-sectional baseline description of 99 adults with type 1 diabetes and problematic hypoglycaemia despite structured education in flexible insulin therapy. The following measures were included: Hypoglycaemia Fear Survey II (HFS-II); Attitudes to Awareness of Hypoglycaemia questionnaire (A2A); Hospital Anxiety and Depression Index; and Problem Areas In Diabetes. *k*-mean cluster analysis was applied to HFS-II and A2A factors. Data were compared with a peer group without problematic hypoglycaemia, propensity-matched for age, sex and diabetes duration (*n* = 81).

**Results:**

The HARPdoc cohort had long-duration diabetes (mean ± SD 35.8 ± 15.4 years), mean ± SD Gold score 5.3 ± 1.2 and a median (IQR) of 5.0 (2.0–12.0) severe hypoglycaemia episodes in the previous year. Most individuals had been offered technology and 49.5% screened positive for anxiety (35.0% for depression and 31.3% for high diabetes distress). The cohort segregated into two clusters: in one (*n* = 68), people endorsed A2A cognitive barriers to hypoglycaemia avoidance, with low fear on HFS-II factors; in the other (*n* = 29), A2A factor scores were low and HFS-II high. Anxiety and depression scores were significantly lower in the comparator group.

**Conclusions/interpretation:**

The HARPdoc protocol successfully recruited people with treatment-resistant problematic hypoglycaemia. The participants had high anxiety and depression. Most of the cohort endorsed unhelpful health beliefs around hypoglycaemia, with low fear of hypoglycaemia, a combination that may contribute to persistence of problematic hypoglycaemia and may be a target for adjunctive psychological therapies.

**Graphical abstract:**

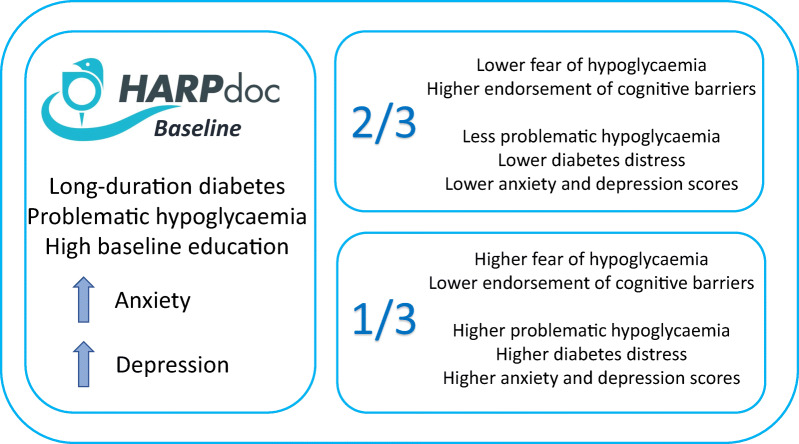

**Supplementary Information:**

The online version contains peer-reviewed but unedited supplementary material available at 10.1007/s00125-022-05679-5.

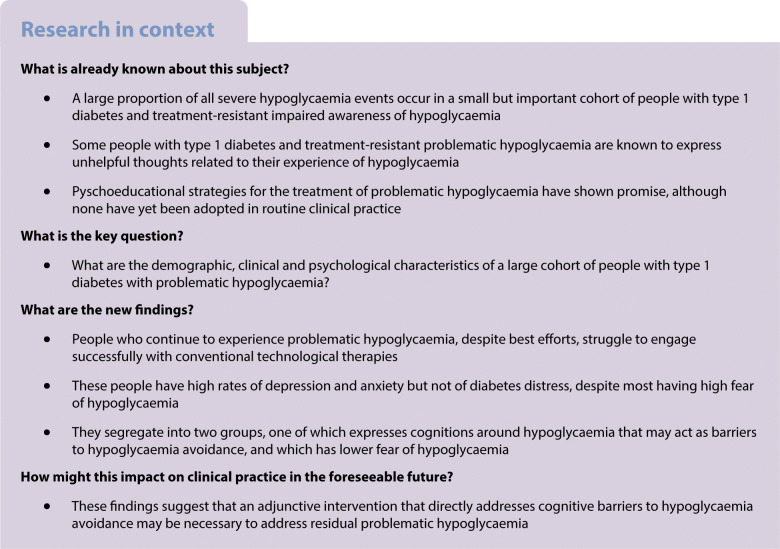



## Introduction

Severe hypoglycaemia (SH) is defined as an episode in which circulating glucose falls to a level too low to sustain normal cognitive function and self-treatment is not possible [1]. SH remains a feared complication of insulin therapy for insulin-deficient diabetes. Recent publications reported that 12% of people with type 1 diabetes experienced one or more SH episode(s) over a 7–8 month follow-up period, with over 4% admitted to hospital for it [2], and recent data from the USA type 1 diabetes exchange registry showed that 5–10% of people reported SH-related coma or seizure [3]. The prevalence of SH increases both with diabetes duration [4] and with impaired awareness of hypoglycaemia (IAH) [5]. IAH is a state in which people are often unable to recognise hypoglycaemia because of delayed and diminished symptomatic and neuroendocrine responses to a falling plasma glucose. IAH increases the risk of SH sixfold in type 1 diabetes [6].

The combination of IAH and recurrent SH is referred to as ‘problematic hypoglycaemia’ [7]. It impairs quality of life, may contribute to loss of driving privileges and places a burden on social, work and family life, as well as increasing the risk of emergency department visits and healthcare costs [8–12].

Structured education in insulin dose adjustment and technological advances in glucose monitoring and insulin delivery have brought about substantial reduction in SH in clinical trials [13], enabling creation of an evidence-based treatment pathway [7]. However, population-level improvements in SH rates, which may have been rising prior to availability of CGM [14], remain too high [2, 3]. The distribution of SH has always been very skewed [5] and some individuals with type 1 diabetes continue to experience SH despite optimised education and technology utilisation [3, 15]. These individuals contribute disproportionately to the overall rates of hypoglycaemia, remain poorly categorised, are frequently excluded from clinical trials, and represent a major unmet need in diabetes clinical care.

A better understanding of the psychology underpinning problematic hypoglycaemia is emerging. In a large unselected cohort of people with type 1 diabetes, those at high risk of SH have been categorised into different levels of personal concern about that risk [16]. The group with high SH risk and low fear of hypoglycaemia (8% of the total and one-third of those experiencing SH) may have reduced ability to avoid hypoglycaemia, as they do not prioritise it. People with IAH have shown reduced likelihood of behaviour change following clinical review in comparison with their peers with intact awareness [17]. Qualitative studies in people with IAH have identified unhelpful thoughts or ‘thinking traps’ in relation to their experience of hypoglycaemia [18]. Using the Attitudes to Awareness of Hypoglycaemia questionnaire (A2A), these thinking traps have been grouped into categories: ‘asymptomatic hypoglycaemia normalised’; ‘hypoglycaemia concern minimised’; and ‘hyperglycaemia avoidance prioritised’ [19].

Psychological principles inform many of the structured education programmes that most successfully reduce the incidence of SH [20–22], although none have eliminated the problem or been tested for their impact on SH and IAH in people with persistent SH after other interventions. The Hypoglycaemia Awareness Restoration Programme for people with type 1 diabetes and problematic hypoglycaemia persisting despite otherwise optimised control (HARPdoc) is a novel 6 week psychoeducational programme developed specifically to address the thinking traps associated with persistence of IAH in people who had completed other structured education in insulin self-management proven to reduce SH and also had access to technological support [23]. The clinical and cost-effectiveness of HARPdoc is currently being compared against Blood Glucose Awareness Training (BGAT) [20] in an international, multicentre, pragmatic, parallel-arm RCT. The cohort recruited has given us the opportunity to study IAH in more detail and advance our understanding of its psychology.

This paper presents the baseline characteristics of the HARPdoc cohort to answer the following primary research questions: (1) what are the demographic, clinical and psychological characteristics of a large cohort of people with type 1 diabetes with problematic hypoglycaemia; and (2) are there clinical and psychological differences in subgroups of participants clustered according to their cognitions and their responses to the Hypoglycaemia Fear Survey II (HFS-II) [24] responses? As a secondary focus of this study, a comparator group of people with type 1 diabetes but without problematic hypoglycaemia was recruited to explore specific characteristics associated with the experience of problematic hypoglycaemia. The third aim of the study was to compare the population characteristics between the HARPdoc cohort and a matched peer group of people without problematic hypoglycaemia.

## Methods

### Recruitment

**Fig. 1:**
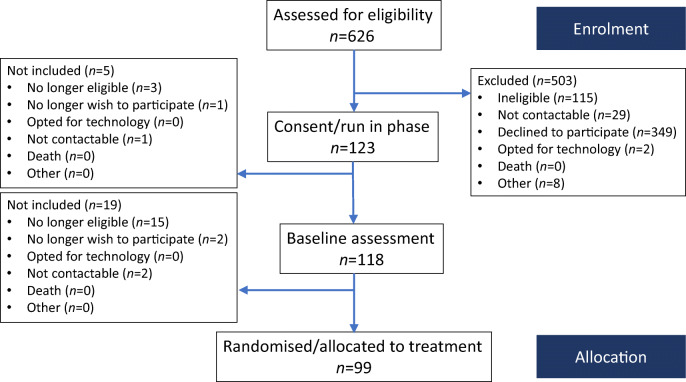
Study flowchart up to randomisation. Those (*n* = 626) assessed for eligibility were people with diabetes who were judged as potential participants by clinicians. In one centre (*n* = 329) this was performed from the electronic patient record followed by cold-calling

The HARPdoc RCT protocol has been published [23]. The trial recruited participants across three specialist diabetes centres in the UK and one in the USA between March 2017 and March 2019. Figure 1 shows the flow of participants to recruitment. Eligible participants were adults with type 1 diabetes of at least 4 years duration, who had experienced problematic hypoglycaemia for at least 1 year, despite having participated in structured education and currently using a flexible insulin regimen. Problematic hypoglycaemia was defined as a Gold or Clarke score ≥4 [25, 26] and more than one episode of SH in the preceding 2 years, with at least one since being on current treatment. Eligible participants were willing to comply with the study design, including performance of glucose self-monitoring at least four times per day, able to communicate in written and spoken English and give written informed consent. The main exclusion criteria were as follows: type 2 diabetes; type 1 diabetes with preserved awareness of hypoglycaemia; no prior attendance at a structured diabetes education programme; currently awaiting islet or whole organ pancreas transplantation; current pregnancy; severe mental disorders including eating disorders; and cognitive impairment independent of hypoglycaemia and presence of an untreated comorbid medical disease other than diabetes that might be expected to contribute to hypoglycaemia risk.

During the screening process, demographic data, medical history and vital signs were taken from eligible, consented participants. Baseline assessment was undertaken immediately prior to randomisation, once a full therapy group had been recruited and the course was ready to begin. Measures collected at the screening and baseline assessment stages form the set of pre-randomisation measures reported in this paper.

For a comparator group, we recruited a second cohort of adults with type 1 diabetes at the UK HARPdoc sites. Consecutive individuals attending for diabetes clinical consultations at the recruiting sites were assessed for the HARPdoc inclusion and exclusion criteria but did not have IAH (Gold or Clarke score ≤3). These participants formed the Cognitions Outcomes and Behaviours around hypoglycaemia in Adults with type 1 diabetes (COBrA) cohort.

The study was approved by the London Dulwich and the Wales Research Ethics Committees (IRAS numbers 216381 and 271164) and the Institutional Review Board of the Joslin Diabetes Center.

### Data collection

Recruitment into the HARPdoc RCT was completed prior to March 2020 and COVID-19 restrictions; visits were face to face and used paper questionnaires. Nineteen of the 81 hypoglycaemia-aware participants of the COBrA comparator study (23.5%) were recruited after March 2020 using virtual platforms, including Qualtrics (www.qualtrics.com) for the questionnaires. Anonymised data were entered into an electronic database (MACRO; Elsevier) by the trial data manager, with the research team blinded to these data.

### Questionnaires

#### SH

Participants were asked to recall episodes of SH over the previous 12 months and, separately, 24 months on a structured form [23]. SH was defined in accordance with the International Hypoglycaemia Study Group definition (https://www.ihsgonline.com/what-is-hypoglycaemia-2/ [accessed 5 Feb 2022]): a hypoglycaemic episode causing cognitive impairment requiring the assistance of another person or inducing seizure or coma. Participants documented the number of episodes that had resulted in the following, over each time frame: loss of consciousness/seizure; parenteral therapy; ambulance call; Accident and Emergency attendance; and overnight hospital admission.

#### HFS-II

The HFS-II is a validated 33-item questionnaire to assess levels of fear around hypoglycaemia [24]. It has two subscales for behaviour (HFS-B, items 1–15) and worry (HFS-W, items 16–33). Participants score each item from 0 (‘never’) to 4 (‘almost always’). Total and subscale scores are reported as the sum of the item rankings. Scores are also reported as mean item scores (total ranking sum score/no. of items in the scale or subscale), to allow comparisons to be made with published data using the original HFS, which has fewer items than the HFS-II. In data kindly supplied by T. Anderbro [16] using the HFS-I survey in an unselected clinic population of 764 people, with ten behaviour and 27 worry items [27], the median of the behaviour subscale was 1.85 and for the worry subscale was 0.92 and values below these were taken to indicate low scores in the present data. While use of a single study to generate thresholds may introduce bias into these estimates, the broad and unselected clinic population of the Anderbro dataset makes it the most robust available source of normative values for the HFS.

#### Attitudes to awareness thinking style

The 19-item A2A questionnaire [19] measures unhelpful health beliefs likely to create cognitive barriers to hypoglycaemia avoidance, the ‘thinking traps’ of impaired awareness. Each item was scored 0 (‘not at all true’) to 3 (‘very true’). Total score and the sum of the items in each of the three thinking styles described in the A2A are reported as follows: ‘asymptomatic hypoglycaemia normalised’ (items 6–7, 10 and 15); ‘hypoglycaemia concern minimised’ (items 11, 14, 17 and 18); and ‘hyperglycaemia avoidance prioritised’ (items 8, 12, 16 and 19). Higher scores indicate greater endorsement of the unhelpful health belief.

#### Problem areas in diabetes

The 20-item Problem Areas In Diabetes (PAID) instrument measures diabetes distress [28]. Each item was scored from 0 (‘not a problem’) to 4 (‘serious problem’). The total score was the sum of all items ×1.25, giving a total score out of 100. A score of 40 or more is indicative of severe distress and reported separately [29].

#### Hospital Anxiety and Depression Scale

The Hospital Anxiety and Depression Scale (HADS) scores 14 items 0–3. It comprises anxiety and depression subscales, where each subscale can score a maximum of 21. The total score in each subgroup is reported, as well as the proportions of people in each group scoring on each subscale as normal (0–7), borderline (8–10) or abnormal (11–21) subgroup scores.

### Biochemistry

HbA_1c_ was measured centrally at the Viapath clinical pathology laboratory at King’s College Hospital, London (HPLC assay, Premier 9210 analyser; Menarini, Italy). HbA_1c_ values for 18.5% of the COBrA comparator group were measured on TOSOH G8 HPLC analysers (Tosoh Bioscience, Japan) and 18.5% using capillary electrophoresis using Sebia capillary 3 tera analysers (Sebia, France). All were run in certified laboratories within the UK’s National Health Service.

### Statistical analysis

A cross-sectional baseline assessment for the HARPdoc cohort was conducted for people with type 1 diabetes and IAH. Demographic, clinical and psychological characteristics are presented as mean ± SD, median (IQR) and frequency (%) as appropriate. Some questionnaire items did not apply to all participants (e.g. ‘to avoid low blood sugar and how it affects me, I limited my driving’ may not be relevant to non-drivers). These items were excluded and for those participants the mean item score reflected the responses to all remaining items.

To categorise the HARPdoc participants by their endorsement of cognitive barriers (A2A scores) as well as their fear of hypoglycaemia, *k*-means clustering was used. The analysis methods are described in detail in electronic supplementary material (ESM) Methods. Differences in the demographic and psychological characteristics of participants in the two clusters were tested and adjusted for multiple comparisons using the Bonferroni correction, which yielded *p*<0.005 to indicate a significant difference.

People in the COBrA cohort were matched 1:1 with the HARPdoc participants using propensity scores, as described more fully in ESM Methods. The final matched dataset of 81 participants from each cohort only included participants who were inside the region of common support, thus excluding participants who had a propensity score so high or low that they did not have a sufficient match. The key sociodemographic and clinical characteristics for the matched participants from both cohorts are presented in a comparison table, and compared using univariate descriptive analysis, to give some preliminary information on relevant characteristics. Following Bonferroni adjustment, *p*<0.002 was taken to indicate a significant difference.

## Results

### Demographic, clinical and psychological characteristics of the cohort of people with type 1 diabetes and with problematic hypoglycaemia

**Fig. 2:**
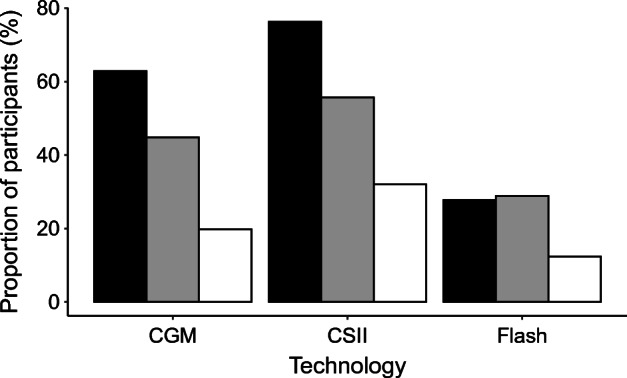
Percentage of participants using technology, including history of offers and usage discontinued. Of the 97 participants, 76.3%, 62.9% and 27.8% had been offered CSII, CGM or Flash, respectively; 55.7%, 44.8% and 28.9% had tried each technology and 32.0%, 19.8% and 12.4% were currently using each technology. Black bars, offered; grey bars, past usage; white bars, current usage

Of 626 people assessed for eligibility, 99 people with type 1 diabetes and IAH were randomised into the HARPdoc RCT (Fig. 1). Most of the people who were ineligible or declined to participate were from a single site at which potential participants were identified by scanning of a large electronic database and then cold-called. Decline rates were much lower when potential participants were identified during clinics or clinical meetings. The demographic, clinical and psychological characteristics of randomised participants are reported in Table 1. They had a long duration of diabetes and 54.5% had experienced problematic hypoglycaemia for over 10 years. Involvement of healthcare resources (expressed per 100 person-years) included 184 glucagon injections, 110 ambulance call outs, 41 presentations to emergency departments and eight inpatient stays. By protocol, all had attended education in flexible insulin therapy, mostly as structured group education using DAFNE (57.9%) (https://dafne.nhs.uk/ [accessed 5 Feb 2022]), BERTIE (16.8%) (https://www.bertieonline.org.uk/ [accessed 5 Feb 2022]), DO-IT (8.4%) (https://www.joslin.org/patient-care/education-programs-and-classes/do-it-program [accessed 5 Feb 2022]) or other (11.6%). Most participants had been offered access to continuous subcutaneous insulin infusion (CSII) (76.3%), continuous glucose monitoring (CGM) (62.9%) and/or intermittently monitored retrospective CGM (flash) (27.8%). At the time of enrolment 56.7% were using some form of diabetes technology (Fig. 2). Seven (7.2%) participants were currently receiving professional psychological support, 35.4% had been offered it in the past and 24.7% had previously accessed it.

**Table 1:** Participant demographics for the complete HARPdoc cohort

Variable	*n*	Total
Age, years	99	54.3 ± 13.3
Female sex, *n* (%)	99	55 (55.6)
White ethnicity, *n* (%)	99	95 (96.0)
BMI, kg/m^2^	98	26.4 ± 4.9
Duration of type 1 diabetes, years	99	35.8 ± 15.4
Duration of problematic hypoglycaemia ≥10 years, *n* (%)	99	54 (54.5)
Education, *n* (%)		
Structured education in flexible insulin self-management	95	84 (88.4)
Other education (e.g. one to one)	95	11 (11.6)
Symptomatic (peripheral) neuropathy, *n* (%)	99	21 (21.2)
Retinopathy, *n* (%)	98	71 (72.4)
Other significant medical conditions, *n* (%)		
CVD	99	46 (46.5)
Chronic kidney disease	99	8 (8.1)
Endocrine disorder	98	43 (43.9)
Diagnosis of epilepsy	98	3 (3.1)
Psychiatric disorder	99	24 (24.2)
Glycaemic variables		
Hypoglycaemia awareness, Gold score	99	5.3 ± 1.2
Hypoglycaemia awareness, modified Clarke score	99	5.4 ± 1.1
SH events in previous 12 months	98	5.0 (2.0–12.0)
SH events in previous 12 months	98	29.5 ± 87.2
SH events in previous 24 months	97	8.0 (3.0–24.0)
SH events in previous 24 months	97	58.7 ± 187.3
Moderate hypoglycaemia episodes in last 4 weeks	95	7.0 (3.0–15.0)
Central HbA_1c_, mmol/mol	98	57.3 ± 13.1
Central HbA_1c_, %	98	7.4 ± 1.2

Data are presented as mean ± SD, median (IQR) or *n* (%)

By design, participants had IAH by Gold and Clarke scores and a high rate of SH (Table 1). The number of SH episodes in the previous year had a skewed distribution (mean, median: 29.5, 5.0). Scores for the study questionnaires (fear of hypoglycaemia, cognitions around hypoglycaemia avoidance, diabetes distress, anxiety and depression) are reported in Table 2. In total, 49.5% had borderline abnormal or abnormal anxiety scores, 35.0% had borderline abnormal or abnormal depression scores and 31.3% had high diabetes distress scores. One-fifth of the population expressed low worry (HFS-W <0.92).

**Table 2:** Scale measures for the complete HARPdoc cohort

Variable	*n*	Total score	Mean item score
HFS-II, total score	97	51.2 ± 26.1	1.6 ± 0.8
HFS-II, behaviour	97	21.1 ± 11.9	1.4 ± 0.8
HFS behaviour score <1.85, *n* (%)	97	73 (75.3)	
HFS-II, worry	97	30.2 ± 16.3	1.7 ± 0.9
HFS worry score <0.92, *n* (%)	97	20 (20.6)	
HFS factor, ran high	97	6.2 ± 3.6	1.6 ± 0.9
HFS factor, sought safety	97	13.8 ± 8.0	1.7 ± 1.0
HFS factor, felt restricted	93	6.5 ± 5.9	1.1 ± 1.0
HFS factor, worry	96	20.2 ± 12.2	1.6 ± 1.0
A2A, total score	97	10.0 ± 5.4	0.8 ± 0.5
A2A, hyperglycaemia avoidance prioritised	97	5.9 ± 2.7	1.5 ± 0.7
A2A, hypoglycaemia concern minimised	97	2.5 ± 1.9	0.6 ± 0.5
A2A, asymptomatic hypoglycaemia normalised	97	1.7 ± 2.2	0.4 ± 0.6
PAID score	96	31.9 ± 20.1	
≥40 (severe diabetes distress), *n* (%)	96	30 (31.3)	
HADS-A score	97	7.5 ± 4.6	
0–7 (normal), *n* (%)	97	49 (50.5)	
8–10 (borderline abnormal), *n* (%)	97	22 (22.7)	
11–21 (abnormal), *n* (%)	97	26 (26.8)	
HADS-D score	97	5.9 ± 4.3	
0–7 (normal), *n* (%)	97	63 (64.9)	
8–10 (borderline abnormal), *n* (%)	97	20 (20.6)	
11–21 (abnormal), *n* (%)	97	14 (14.4)	
HARPdoc clusters			
High fear/low barriers, *n* (%)	97	29 (29.9)	
Low fear/high barriers, *n* (%)	97	68 (70.1)	

Data are presented as mean ± SD or *n* (%)

### Clustering of HARPdoc participants by A2A and HFS-II responses

**Fig. 3:**
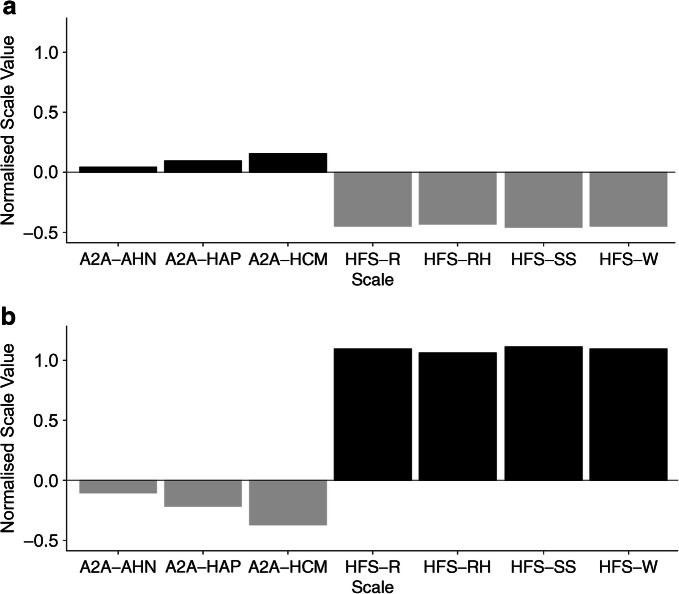
*k*-means clustering analysis of the HARPdoc cohort with two clusters. Black bars, positive endorsement of factors (higher scores); grey bars, negative endorsement (low scores). (**a**) Factor endorsement scores for the high barriers/low fear cluster (*n* = 68). (**b**) Factor endorsement scores for the low barriers/high fear cluster (*n* = 29). AHN, HAP and HCM represent factors of the A2A; R, RH, SS and W represent factors of the HFS. AHN, asymptomatic hypoglycaemia normalised; HAP, hyperglycaemia avoidance prioritised; HCM, hypoglycaemia concern minimised; R, restricted; RH, ran high; SS, sought safety; W, worry

The results of the *k*-means cluster analysis are shown in Fig. 3. A two-cluster model was chosen as having the best fit (ESM Fig. 1) with no overlap. One cluster (*n* = 68 [70%]) was characterised by high scores for the A2A factors (barriers) and low scores for HFS-II factors (fear) and the other (*n* = 29 [30%]) by low scores for barriers and high scores for fear. Key characteristics of the two clusters are summarised in Table 3 and Fig. 3. The clusters were not different in demography, hypoglycaemia awareness, type 1 diabetes duration or use of technology. The larger cluster, endorsing the cognitive barriers and with low fear, had less anxiety (HADS anxiety subscale [HADS-A] mean 6.2 vs 10.7, *p*<0.001) and depression (HADS depression subscale [HADS-D] mean 4.7 vs 8.8, *p*<0.001) and less diabetes related distress (PAID mean 25.4 vs 47.5, *p*<0.001).

**Table 3:** Key characteristics by cluster

Variable	Low fear/high barriers		High fear/low barriers		*p* value^a^
	*n*	Variable measure	*n*	Variable measure	
Age, years	68	54.3 ± 13.9	29	54.5 ± 12.5	0.931
Female sex, *n* (%)	68	35 (51.5)	29	18 (62.1)	0.337
Diabetes duration, years	68	35.2 ± 15.1	29	36.4 ± 16.2	0.712
SH in previous 12 months	68	4.0 (1.5–8.0)	29	9.0 (5.0–30.0)	0.003*
Gold score	68	5.0 ± 1.1	29	6.0 ± 1.0	<0.001*
(Modified) Clarke score	68	5.2 ± 1.2	29	5.7 ± 0.7	0.052
Use of technology					
Any technology (pump/CGM/pump with automated suspend), *n* (%)	68	40 (58.8)	29	15 (51.7)	0.518
Retrospective intermittently monitored ‘flash’ glucose monitoring, *n* (%)	68	9 (13.2)	29	3 (10.3)	0.491
PAID score	68	25.4 ± 16.2	28	47.5 ± 20.5	<0.001*
PAID ≥40 (severe diabetes distress), *n* (%)	68	13 (19.1)	28	17 (60.7)	
HADS-A score	68	6.2 ± 4.0	29	10.7 ± 4.5	<0.001*
0–7 (normal), *n* (%)	68	41 (60.3)	29	8 (27.6)	
8–10 (borderline abnormal), *n* (%)	68	16 (23.5)	29	6 (20.7)	
11–21 (abnormal), *n* (%)	68	11 (16.2)	29	15 (51.7)	
HADS-D score	68	4.7 ± 3.3	29	8.8 ± 5.0	<0.001*
0–7 (normal) *n* (%)	68	52 (76.5)	29	11 (37.9)	
8–10 (borderline abnormal), *n* (%)	68	13 (19.1)	29	7 (24.1)	
11–21 (abnormal), *n* (%)	68	3 (4.4)	29	11 (37.9)	

Data are presented as mean ± SD, median (IQR) or *n* (%)

^a^*p* value from a two-sample independent *t* test where mean ± SD is presented, Mann–Whitney two-sample statistic where median (IQR) is presented, and χ^2^ test statistic (Fisher’s Exact where cell frequencies <5) where *n* (%) is presented

*p<0.005; considered statistically significant following Bonferroni correction

### Comparison of HARPdoc participants with non-problematic hypoglycaemia peer group

One hundred and six people consented to COBrA and 81 returned completed questionnaire booklets. The propensity score matching algorithm identified 81 participants (HARPdoc_81_ and COBrA_81_) in each cohort matched for diabetes duration and sex (Table 4). By design, the groups had differences in the Gold and Clarke scores and SH rates. HbA_1c_ was not significantly different, nor were there differences in macro- or microvascular complications and comorbidities between the cohorts. As shown in Table 4, participants in the HARPdoc_81_ group had higher HFS-II scores for total score and behaviour subscale and worry subscale scores. Self-report of psychiatric comorbidities was higher in the HARPdoc_81_ group, predominantly depression and anxiety, as was prevalence of both anxiety and depression measured by HADS subscales. A higher mean PAID score, reflecting diabetes distress, did not achieve significance after correction for multiple comparisons.

**Table 4:** Comparison of baseline characteristics in the matched dataset of HARPdoc (*n* = 81) and COBrA (*n* = 81) participants

Variable	HARPdoc		COBrA		*p* value^a^
	*n*	Variable measure	*n*	Variable measure	
Demographics					
Age, years	81	51.8 ± 13.2	81	48.0 ± 14.1	0.086
Female sex, *n* (%)	81	46 (56.8)	81	47 (58.0)	0.874
Diabetes duration, years	81	30.9 ± 12.1	81	30.2 ± 11.9	0.727
Gold score	81	5.3 ± 1.2	81	1.7 ± 0.6	<0.001*
(Modified) Clarke score	81	5.4 ± 1.0	79	1.6 ± 0.7	<0.001*
SH in previous 12 months^b^	81	5.0 (2.0–10.0)	81	0.0 (0.0–0.0)	<0.001*
HbA_1c_, mmol/mol	81	58.2 ± 13.1	68	63.4 ± 11.1	0.022
HbA_1c_,%	81	7.5 ± 1.2	68	7.9 ± 0.9	
Symptomatic peripheral neuropathy, *n* (%)	81	17 (21.0)	79	6 (7.6)	0.016
Retinopathy, *n* (%)	80	57 (71.3)	81	53 (65.4)	0.428
Psychiatric condition, *n* (%)	81	20 (24.7)	72	2 (2.8)	<0.001
Diabetes technology in use					
Pump, *n* (%)	80	24 (30.0)	66	23 (34.8)	0.533
Pump with automated suspend feature, *n* (%)	79	14 (17.7)	53	3 (5.7)	0.062
CGM, *n* (%)	80	14 (17.5)	59	10 (16.9)	0.932
Any technology (pump/CGM/pump with automated suspend), *n* (%)	80	44 (55.0)	66	32 (48.5)	0.433
Retrospective intermittently monitored ‘flash’ glucose monitoring, *n* (%)	80	8 (10.0)	65	30 (46.2)	<0.001*
Support from a diabetes psychologist, psychiatrist or counsellor, *n* (%)	80	7 (8.8)	61	3 (4.9)	0.514
Clinical scales					
HFS-II, mean item score	80	1.6 ± 0.8	67	0.9 ± 0.4	<0.001*
HFS-II, mean behaviour item score	80	1.4 ± 0.8	67	0.9 ± 0.4	<0.001*
HFS-II, mean worry item score	80	1.7 ± 0.9	67	0.9 ± 0.6	<0.001*
A2A, total score	80	9.8 ± 5.4	65	9.4 ± 3.9	0.569
A2A, hyperglycaemia avoidance prioritised	80	5.7 ± 2.7	65	5.0 ± 2.3	0.100
A2A, hypoglycaemia concern minimised	80	2.3 ± 1.8	65	2.7 ± 1.9	0.207
A2A, asymptomatic hypoglycaemia normalised	80	1.8 ± 2.2	65	1.6 ± 1.8	0.705
PAID score	80	33.3 ± 20.2	67	23.8 ± 17.5	0.003
PAID ≥40 (severe diabetes distress), *n* (%)	80	27 (33.8)	67	14 (20.9)	
HADS-A score	80	8.0 ± 4.5	67	5.7 ± 3.3	<0.001*
HADS-A 11–21 (abnormal), *n* (%)	80	23 (28.8)	67	7 (10.4)	
HADS-D score	80	6.3 ± 4.4	67	3.6 ± 2.9	<0.001*
HADS-D 11–21 (abnormal), *n* (%)	80	14 (17.5)	67	2 (3.0)	

Data are presented as mean ± SD, median (IQR) or *n* (%)

^a^*p* value from a two-sample independent *t* test where mean ± SD is presented, Mann–Whitney two-sample statistic where median (IQR) is presented, and χ^2^ test statistic (Fisher’s Exact where cell frequencies <5) where *n* (%) is presented

^b^Data taken from anonymous questionnaires for HARPdoc_81_ and from similar but open questionnaires for COBrA_81_

*p<0.002; considered statistically significant following Bonferroni correction

There was little difference in the utilisation of technology, although people in the COBrA_81_ cohort more frequently used flash glucose monitoring, likely reflecting the relative availability of this device when HARPdoc recruitment occurred.

## Discussion

Our data show that the HARPdoc trial successfully recruited a cohort of people with type 1 diabetes, IAH and recurrent SH that has persisted despite optimised conventional self-management of their insulin regimens. The cohort had long diabetes duration and high scores on screening for anxiety and depression but did not have high levels of diabetes distress. The rate of SH was high, with a median of five SH events in the previous year, demonstrating the high-risk nature of the participants enrolled. However, the rate was very skewed, with a mean of 30 events. This wide variation in rates of SH suggests heterogeneity in the population, and we also noted heterogeneity in the fears, behaviours and cognitions related to hypoglycaemia. Most notably, the group segregated into individuals who endorsed attitudinal barriers to hypoglycaemia avoidance and had relatively low levels of fear and another group that had low scores on attitudinal barriers and expressed relatively high fear. A comparison between HARPdoc participants and a cohort of people matched for diabetes duration and sex but without problematic hypoglycaemia showed that the HARPdoc cohort overall exhibited higher worry about hypoglycaemia, although higher mean score for diabetes distress did not survive correction for multiple comparisons. The comparison analysis confirmed the high rates of anxiety and depression in the HARPdoc cohort. The two groups did not show significant differences when comparing attitudinal barriers to hypoglycaemia avoidance.

This HARPdoc cohort represents one of the largest datasets of people with type 1 diabetes and treatment-resistant problematic hypoglycaemia and therefore provides a unique opportunity to examine the clinical and psychological characteristics of this group in detail. Higher levels of general anxiety and depression, as measured by the HADS, suggests a group with higher psychological comorbidity that may predispose to or exacerbate struggles with management of their problematic hypoglycaemia and a desire to avoid hyperglycaemia. Whether the anxiety and depression are driven by the problematic hypoglycaemia cannot be determined from these data.

As expected for people with IAH and high rates of severe episodes, the cohort has long diabetes duration. The duration of problematic hypoglycaemia was also long (>10 years in over half of the participants), suggesting a level of unmet need within diabetes services. This raises questions about the efficacy of traditional medical interventions for this population. All the participants were attending specialist type 1 diabetes services, which offer delivery of the recommended treatments for hypoglycaemia [7]. Indeed, completion of structured education in flexible insulin therapy known to minimise rates of SH [30] was an inclusion criterion. Most participants had been offered at least CSII therapy and/or CGM and over one-quarter had been offered sensor augmented pump therapy as a second-line intervention, a high percentage given that recruitment occurred between 2017 and 2019 [3]. While more recent improvements in diabetes technology, such as hybrid closed loop, might provide this cohort with better protection, it remains the case that only half used some form of technology and many who did try various devices discontinued the technology by the time of recruitment. In the comparison with the COBrA cohort, no more of the HARPdoc group were using technology than the group untroubled by hypoglycaemia, despite clinical guidelines recommending prioritising its use for people with problematic hypoglycaemia [31]. Given earlier evidence for reduced engagement with healthcare-recommended therapeutic strategies in people with IAH [17], this disengagement with technology and persistence of problematic hypoglycaemia points to drivers of hypoglycaemia beyond traditional pharmacological and pathophysiological mechanisms. Meanwhile, recent data show the persistence of SH in 12% of people at low risk using retrospective intermittent CGM and 25% of people at high risk using real-time CGM. Less successful outcomes with technology are compatible with our hypothesis that there is an important group of people whose problematic hypoglycaemia fails to respond adequately even when technology is in place and who require additional approaches [2, 32].

In previous studies, people with IAH have described health beliefs related to their hypoglycaemia that likely constitute barriers to hypoglycaemia avoidance [18, 19]; these have been postulated as contributors to the development and maintenance of IAH and recurrent SH [18, 33, 34]. Low fear [16] or ‘lack of concern’ [18] have been described in some people with IAH and experience of SH, with low concern occurring in about one-third of people at high risk for SH in the study by Anderbro et al [16]. In the HARPdoc cohort, the total HFS-II score was significantly higher than in the hypoglycaemia-aware COBrA group, although one-fifth of the participants with IAH had low worry about hypoglycaemia. Nevertheless, the data indicate that most of the HARPdoc participants were concerned about their hypoglycaemia when compared with the median values from a single reference study [16] and they may have exhibited more diabetes distress. In the present data, the absolute scores for the ‘thinking traps’ (the A2A factors) were not different between HARPdoc participants and those with intact awareness. One possibility is that some of the A2A questions are less relevant to people who have intact hypoglycaemia awareness, leading to unexpected answers in ‘asymptomatic hypoglycaemia normalised’, while we might expect that people in both cohorts may endorse cognitions associated with ‘hyperglycaemia avoidance prioritised’. Additionally, It may be that unhelpful beliefs are necessary but not sufficient to drive problematic behaviours and that the presence of other mediating factors, such as certain personality traits, can affect how beliefs influence behaviour [35].

To examine this further, a clustering analysis was conducted to investigate the interaction between cognitive barriers (A2A factors) and fear of hypoglycaemia (HFS-II factors) in the HARPdoc cohort. The people in one cluster endorsed cognitive barriers to hypoglycaemia avoidance, with above average scores on A2A factors, but had lower scores in the HFS-II factors (‘high barriers/low fear’). In contrast, people in the second cluster showed the reverse pattern, with low endorsement of the cognitive barriers and higher fear of hypoglycaemia scores (‘low barriers/high fear’). The two groups did not differ in age, diabetes duration, technology use or their responses to the hyperglycaemia avoidance scale. The main difference between the clusters appeared to be in the lower HADS anxiety and depression scores in the low fear/high barriers group. We speculate that it is people in this cluster who are likely to benefit from, and perhaps even need, therapies that directly target the cognitive ‘thinking traps’. The HARPdoc trial outcome data will allow us to explore this hypothesis. The two clusters differed significantly in Gold score and SH frequency, with the ‘high fear/low barriers’ cluster reporting greater severity of IAH and more SH events in the prior year. We postulate that people with high fear may be strongly motivated towards behavioural change and therefore may benefit from either trial intervention.

Avoidance of hyperglycaemia and complications of diabetes can be a rationale for excessive lowering of blood glucose [18]. The similar rates of retinopathy reported in the HARPdoc and COBrA cohorts may indicate that this strategy is not always effective in reducing microvascular complications, perhaps related to the lack of significant difference in HbA_1c_ and also perhaps because of stress and inflammation related to hypoglycaemia or higher glycaemic variability [36, 37]. The cross-sectional nature of these observations makes it impossible to ascribe causality; however, in the HARPdoc cohort problematic hypoglycaemia appears to occur frequently as part of a constellation of medical and psychiatric conditions. It will be important to look for evidence of improvement in mood and anxiety disorders as part of the anticipated recovery from IAH or recurrent SH in the HARPdoc trial.

The strengths of the present study include the number of participants recruited as having high burden of SH and significant impairment in symptomatic awareness of hypoglycaemia. This has allowed us to examine psychological variables that characterise individuals in this population. The cohort of people without problematic hypoglycaemia but matched for diabetes duration and sex, two major influences on hypoglycaemia risk, provided a robust comparator group.

There are limitations to this study. First, as a cross-sectional analysis of a trial population we may have recruited a biased sample willing to participate in a research study. We also recognise that recruitment targets for the HARPdoc RCT were based on a power calculation to detect differences in the outcomes of participants undertaking either HARPdoc or BGAT. Despite this, evidence suggests that our cluster sizes are sufficient to make accurate assessments [38]. We also acknowledge that self-reported SH events may be subject to potential recall bias. This is mitigated by evidence for accurate recall of SH over 12 months and the use of anonymised reporting [39].

To conclude, this study describes in detail the demographic, medical and psychological characteristics of the participants in the HARPdoc study by virtue of their type 1 diabetes and treatment-resistant problematic hypoglycaemia. As a group, they experience a high burden of anxiety and depression. They can be broadly divided into two subgroups described as having high barriers/low fear and low barriers/high fear to hypoglycaemia avoidance and hypoglycaemia itself, respectively. The heterogeneity in emotional, cognitive and behavioural characteristics supports the need for complex and flexible interventions that can address numerous barriers to improving hypoglycaemia symptom awareness and hypoglycaemia risk. The HARPdoc trial will allow us to examine whether the identified mental health issues resolve with a unique intervention targeting cognitions and whether holding certain cognitive barriers to hypoglycaemia avoidance with low fear of hypoglycaemia predicts the need for a cognitive intervention such as HARPdoc.

## Supplementary information

ESM (PDF 151 kb)

## Data Availability

The datasets generated during and/or analysed during the current study are available from the corresponding author on reasonable request.

## Abbreviations

A2A: Attitudes to Awareness of Hypoglycaemia questionnaire
BGAT: Blood glucose awareness training
CGM: Continuous glucose monitoring
COBrA: Cognitions Outcomes and Behaviours around hypoglycaemia in Adults with type 1 diabetes
CSII: Continuous subcutaneous insulin infusion
HADS: Hospital Anxiety and Depression Scale
HADS-A: HADS anxiety subscale
HADS-D: HADS depression subscale
HARPdoc: Hypoglycaemia Awareness Restoration Programme for people with type 1 diabetes and problematic hypoglycaemia persisting despite optimised control
HFS-II: Hypoglycaemia Fear Survey II
IAH: Impaired awareness of hypoglycaemia
PAID: Problem Areas In Diabetes
SH: Severe hypoglycaemia
