# Supplementary material for: Characteristics of adults with type 1 diabetes and treatment-resistant problematic hypoglycaemia: a baseline analysis from the HARPdoc RCT
Source: Diabetologia. 2022 Mar 24;65(6):936–48. doi: 10.1007/s00125-022-05679-5 (PMC8943518; doi:10.1007/s00125-022-05679-5)
Supplement: Supplementary file 1 — (PDF 151 kb) [file 125_2022_5679_MOESM1_ESM.pdf]

ESM Methods

Cluster analysis of cognitions and fear concerning hypoglycaemia in participants in the HARPdoc RCT

In order to characterise the HARPdoc participants by their scores on the Attitudes to Awareness (A2A) and Hypoglycaemia Fear (HFS-II) questionnaires, K-means clustering was performed in R (version 3.6.1). HFS-II responses were first partitioned according to a factor analysis that had been conducted across both HARPdoc and COBrA cohorts [1]. Mean item scores for these factors, and for A2A factors, were weighted according to the question eigenvalue from the original factor analyses [1, 2]. The factor scores were the mean of the weighted item responses within each factor. This weighting step ensured that clustering occurred according to the responses most closely aligned with each factor. Individual scores for each factor were then normalised by subtracting the mean score and dividing by the SD. The optimal number of clusters was identified with a silhouette analysis using the *factoextra* package. The silhouette method was chosen as an objective measure of cluster cohesion (Figure S1). Stability validation was conducted using the *clValid* package which calculated several stability measures, the average proportion of non-overlap, the average distance, the average distance between means and the figure of merit. These stability measures provided inconsistent results (2-10 clusters) and K-mean clustering was then performed using the optimal number of clustering centres (two; SF1) from the Silhoutte analysis as this matched our theoretical basis. We used the Hartigan and Wong (1979) algorithm as implemented in *kmeans* [3]. This algorithm uses the Euclidean distance as the similarity measure.

Propensity matching methodology

Stata command *psmatch2* firstly estimated the propensity score of the cohort membership (HARPdoc vs hypoglycaemia aware COBrA participants) based on specified covariates gender and duration of diabetes, selected as known demographic factors that influence hypoglycaemia experience [4, 5]. Participants were then matched on a 1:1 basis with no replacements, using a calliper set to 0.2 SD of the propensity score (the maximum tolerated difference between matched subjects).

ESM Fig. 1

Output from the silhouette analysis supporting the use of a two-cluster solution.

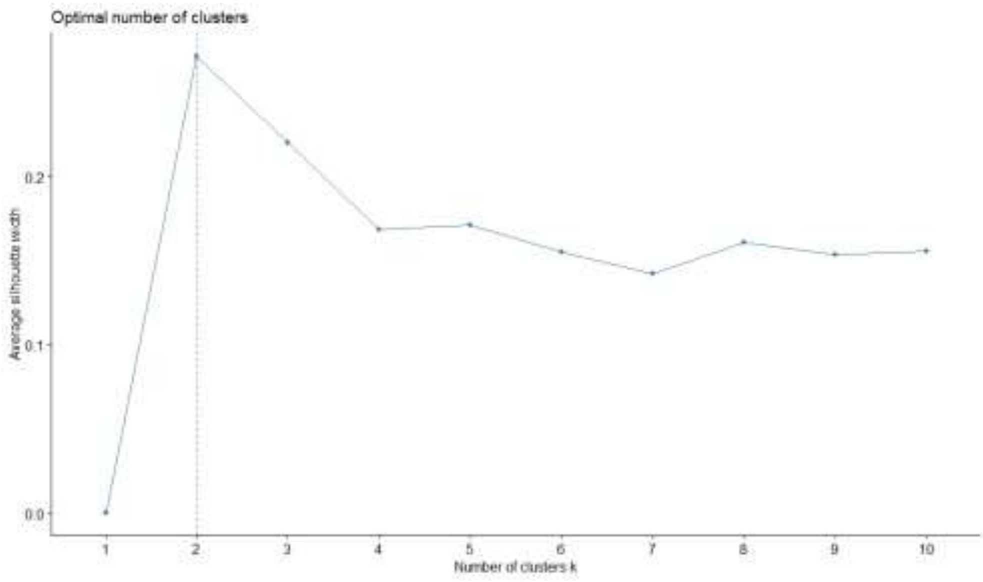

References

1. Maclean RH, Jacob P, Haywood S, et al (2020) 156-OR: Fear of Hypoglycemia in People with Type 1 Diabetes and Problematic Hypoglycaemia: A Mismatch between Worry and Behaviour? In: Diabetes. American Diabetes Association, p 156–OR

2. Cook AJ, DuBose SN, Foster N, et al (2019) Cognitions associated with hypoglycemia awareness status and severe hypoglycemia experience in adults with type 1 diabetes. Diabetes Care 42(10):1854–1864. https://doi.org/10.2337/dc19-0002

3. Hartigan JA, Wong MA (1979) Algorithm AS 136: A K-Means Clustering Algorithm. Appl Stat 28(1):100. https://doi.org/10.2307/2346830

4. Gonder-Frederick LA, Schmidt KM, Vajda KA, et al (2011) Psychometric properties of the hypoglycemia fear survey-ii for adults with type 1 diabetes. Diabetes Care 34(4):801–806. https://doi.org/10.2337/dc10-1343

5. Amiel SA, Maran A, Powrie JK, Umpleby AM, Macdonald IA (1993) Gender differences in counterregulation to hypoglycaemia. Diabetologia 36(5):460–464. https://doi.org/10.1007/BF00402284
